# Interaction Effect of Baseline Serum Glucose and Early Ischemic Water Uptake on the Risk of Secondary Hemorrhage After Ischemic Stroke

**DOI:** 10.3389/fneur.2021.690193

**Published:** 2021-07-08

**Authors:** Jawed Nawabi, Sarah Elsayed, Henriette Scholz, André Kemmling, Lukas Meyer, Helge Kniep, Matthias Bechstein, Fabian Flottmann, Tobias D. Faizy, Gerhard Schön, Jens Fiehler, Uta Hanning, Gabriel Broocks

**Affiliations:** ^1^Department of Diagnostic and Interventional Neuroradiology, University Medical Center Hamburg-Eppendorf, Hamburg, Germany; ^2^Department of Radiology (CCM), Charité - Universitätsmedizin Berlin, Campus Mitte, Humboldt-Universität zu Berlin, Freie Universität Berlin, Berlin, Germany; ^3^Berlin Institute of Health, BIH Biomedical Innovation Academy, Berlin, Germany; ^4^University Medical Center Schleswig-Holstein, Campus Lübeck, Lübeck, Germany; ^5^University Medical Center Marburg, Marburg University, Marburg, Germany; ^6^Institute of Medical Biometry and Epidemiology, University Medical Center Hamburg-Eppendorf, Hamburg, Germany

**Keywords:** glucose, edema, stroke, intracerebral hemorrhage, outcome

## Abstract

**Background and Purpose:** Intracerebral hemorrhage (ICH) after mechanical thrombectomy (MT) for acute ischemic stroke (AIS) remains a major complication and its early prediction is of high relevance. Baseline serum glucose (BGL) is a known predictor of ICH, but its interaction with early ischemic changes remains uncertain. We hypothesized that BGL interacts with the effect of tissue water uptake on the occurrence of ICH.

**Methods:** Three hundred and thirty-six patients with acute ischemic stroke treated with MT were retrospectively analyzed. ICH was diagnosed within 24 h on non-enhanced CT (NECT) and classified according to the Heidelberg Bleeding Classification. Early tissue water homeostasis has been assessed using quantitative lesion net water uptake (NWU) on admission CT. Multivariate logistic regression was used to identify predictors of ICH.

**Results:** One hundred and seven patients fulfilled the inclusion criteria of which 37 (34.6%) were diagnosed with ICH. Patients with ICH had a significant higher BGL on admission (median 177 mg/dl, IQR: 127–221.75, *P* < 0.001). In patients with low BGL (<120 mg/dl), higher NWU was associated with 1.34-fold increased likelihood of ICH, while higher NWU was associated with a 2.08-fold increased likelihood of ICH in patients with a high BGL (>200 mg/dl). In multivariable logistic regression analysis, BGL (OR: 1.02, 95% CI: 1.00–1.04, *P* = 0.01) and NWU (OR: 2.32, 95% CI: 1.44–3.73, *P* < 0.001) were significantly and independently associated with ICH, showing a significant interaction (*P* = 0.04).

**Conclusion:** A higher degree of early tissue water uptake and high admission BGL were both independent predictors of ICH. Higher BGL was significantly associated with accelerated effects of NWU on the likelihood of ICH. Although a clear causal relationship remains speculative, stricter BGL control and monitoring may be tested to reduce the risk of ICH in patients undergoing thrombectomy.

## Introduction

Large randomized controlled trials provide efficacy of mechanical thrombectomy (MT) over medical treatment in patients with acute ischemic stroke (AIS) (1, 2). Irrespective of this success, intracerebral hemorrhage (ICH) remains a common and challenging complication with a negative impact on functional outcome. It is these very trials that have further verified a steady rate of symptomatic intracerebral hemorrhage (sICH) with 4.4% after MT when compared with intravenous thrombolysis (1). In addition, also asymptomatic ICH (aICH) has proven to have a negative impact on long-term functional outcome (3–6). Therefore, evaluating the risk factors for ICH becomes an important issue for continuously improving the efficacy of MT in patients with AIS. In patients with AIS, hyperglycemia has been a long recognized and frequent finding with up to 50% (7, 8) and repeatedly associated with increased bleeding events after thrombolytic therapy (IVT) and poor functional outcome (7–10). Furthermore, a linear relationship between increasing serum glucose and ischemic brain edema has been elucidated (11) and the latter is an independent risk factor for ICH after successful MT. The current state of literature provides no sufficient data for the mutual impact of serum glucose and early ischemic brain edema on the risk of ICH in patients with AIS (12–14). Utilizing the widely accepted Heidelberg Bleeding Classification (15), the objectives of the present study were to analyze the mutual impact of serum glucose and ischemic brain edema on the risk of ICH in patients with AIS after successful MT and, in particular, to investigate whether different glucose levels modify the effect of early edema formation on clinical outcome. We hypothesized that baseline serum glucose levels interact with the effect of early ischemic brain edema on functional outcome.

## Methods

### Patients

**Figure 1:**
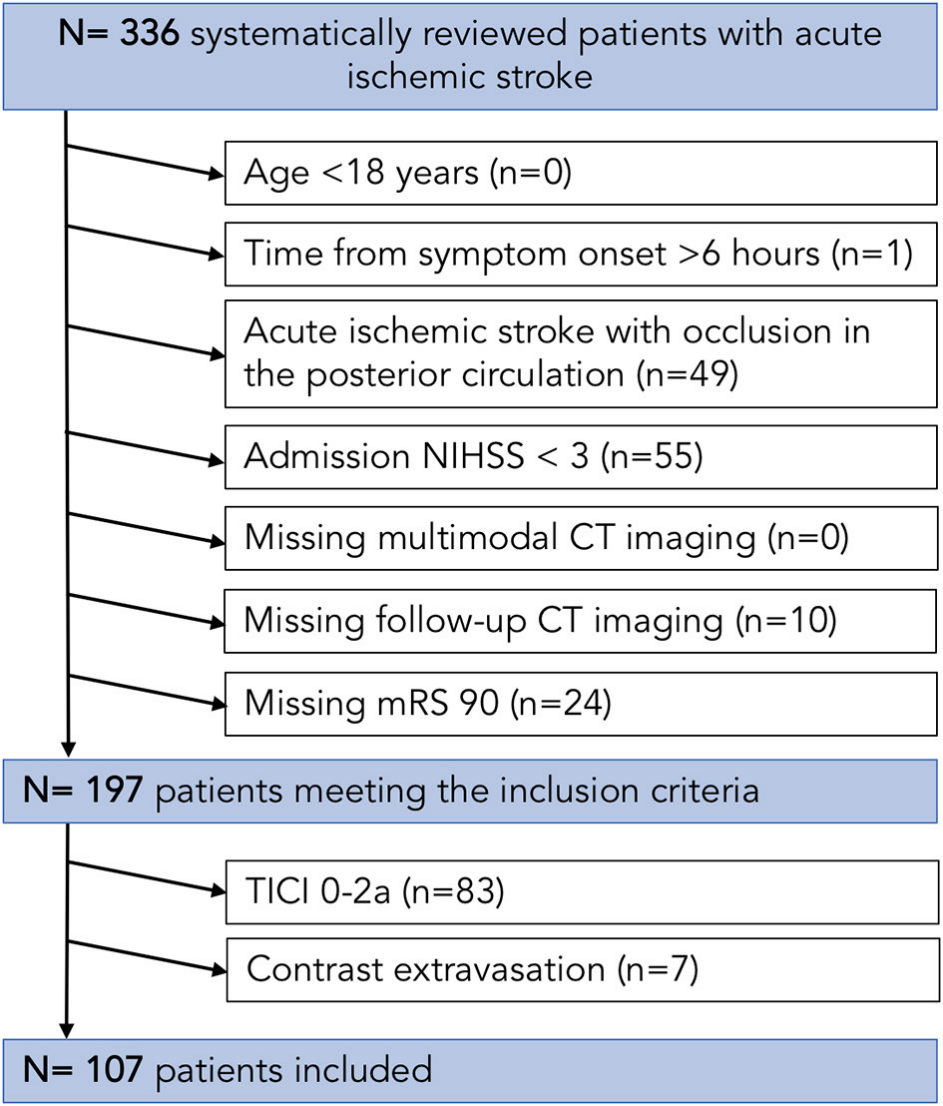
Patient flowchart. CT, computed tomography; NIHSS, National Institutes of Health Stroke Scale (NIHSS); TICI, thrombolysis in cerebral infarction.

Data of an anonymized cohort of 107 consecutive AIS patients with occlusion in the anterior circulation were retrospectively evaluated, in whom successful MT (TICI 2b/3) was performed (Figure 1). Patients were admitted between June 2015 and March 2018 in the University Medical Center Hamburg-Eppendorf (*n* = 336). Data were analyzed after ethical board approval, and informed consent was waived by the institutional review board. The data that support the findings of this study are available from the corresponding author in accordance with the institution's data security regulations upon reasonable request. The patients were screened consecutively based on the following *a priori* defined inclusion criteria: (1) acute ischemic stroke with occlusion of the middle cerebral artery (MCA) or terminal internal carotid artery (ICA), (2) initially performed multimodal CT protocol with CT angiography (CTA) and perfusion CT (CTP), (3) known time from symptom onset to imaging <6 h, (4) follow-up CT (FU-NECT) within 24 h after admission imaging, (5) NIHSS score above 3, (6) documented NIHSS after 24 h and modified Ranking Scale (mRS) after 90 days, and (7) absence of pre-existing thromboembolic or hemodynamic infarctions in admission non-enhanced CT (NECT) within 24 h of stroke symptom onset. Baseline clinical characteristics including baseline serum glucose (BGL) and demographic information were filtered from the medical records. The follow-up CT (FCT) was analyzed for secondary ICH.

### Image Acquisitions

All CT scans were performed on 256 slice scanners (Philips iCT 256) with the following imaging parameters: NECT with 120 kV, 280–320 mA, 5.0 mm slice reconstruction; CTA: 100–120 kV, 260–300 mA, 1.0 mm slice reconstruction, 5 mm MIP reconstruction with 1 mm increment, 0.6-mm collimation, 0.8 pitch, H20f soft kernel, 80 ml highly iodinated contrast medium and 50 ml NaCl flush at 4 ml/s; scan starts 6 s after bolus tracking at the level of the ascending aorta; CTP: 80 kV, 200–250 mA, 5 mm slice reconstruction (max. 10 mm), slice sampling rate 1.50 s (min. 1.33 s), scan time 45 s (max. 60 s), biphasic injection with 30 ml (max. 40 ml) of highly iodinated contrast medium with 350 mg iodine/ml (max. 400 mg/ml) injected with at least 4 ml/s (max. 6 ml/s) followed by 30 ml sodium chloride chaser bolus. All perfusion datasets were inspected for quality and excluded in case of severe motion artifacts.

### Image Analysis

For image analysis, anonymized CT imaging scans were evaluated independently by two radiologists with 5 (JN) and 7 (GB) years of dedicated neuroradiology experience, blinded to all clinical and imaging information except stroke side.

### Collateral Score

CTA collaterals were assessed independently on admission intracranial CTA MIPs and scored according to the grading system of Souza et al. (16) into grades 0 to 4: CS: 0 = absent collaterals in >50% of an MCA M2 branch (superior or inferior division) territory; 1 = diminished collaterals in >50% of an MCA M2 branch territory; 2 = diminished collaterals in <50% of an MCA M2 branch territory; 3 = collaterals equal to the contralateral hemisphere; and 4 = increased collaterals. In addition, collateral grades were grouped into good (collateral score 3–4), partial (collateral score 2), and poor (collateral score 0–1) (14). Calculated Cohen's kappa for interrater reliability was 0.91. Collaterals were dichotomized into poor collaterals as grade 0–2 and good collaterals as 3–4 (14).

### Quantification of Ischemic Brain Edema

Anonymized admission CT imaging scans were segmented semimanually using commercially available software (Analyze 11.0, Biomedical Imaging Resource, Mayo Clinic, Rochester, MN, USA) to derive net water uptake (NWU). The edematous proportion of the hypoattenuated ischemic lesion (% water uptake) was quantified using CT densitometry as previously published (17, 18). In brief, edematous volumetric changes of ischemic lesions due to water uptake were directly quantified by measurements of relative hypoattenuation (Equation 1). Visually evident edematous hypoattenuation was identified as infarct core lesion for further analysis, and a region of interest (ROI) was placed in this infarct core lesion (*D*ischemic). A symmetric ROI was mirrored automatically within the normal tissue of the contralateral hemisphere in order to obtain the density of the normal tissue prior to the infarction (*D*normal) (19–21). CT perfusion was used in addition to aid ROI definition of the early ischemic core by simultaneously presenting cerebral blood volume (CBV) parameter maps at a window between 0 and 6 ml/100 ml (19–22). ROIs were segmented with semiautomatic edge detection and sampled between 20 and 80 HU as described by Broocks et al. (19–22). Inaccuracies were corrected in consensus reading, if necessary. Relative NWU was calculated based on *D*_ischemic_ and *D*normal according to Equation 1.

Equation 1 (19–21):

(1) $$\begin{array}{r} \%\;water\;uptake=\left(1-\frac{Dischemic}{Dnormal}\right)\times 100 \end{array}$$

### Intracranial Hemorrhage Classification

ICH diagnosis and classification was performed on FU-NECT according to the Heidelberg Bleeding Classification (15). All patients with a hyperdense phenomena without mass effect on first follow-up CT at 24 h received a minimum second follow-up CT for evaluation of contrast extravasation. Contrast extravasation was classified with a disappearance with 24 h on second FU-NECT (23).

### Statistical Analysis

**Figure 2:**
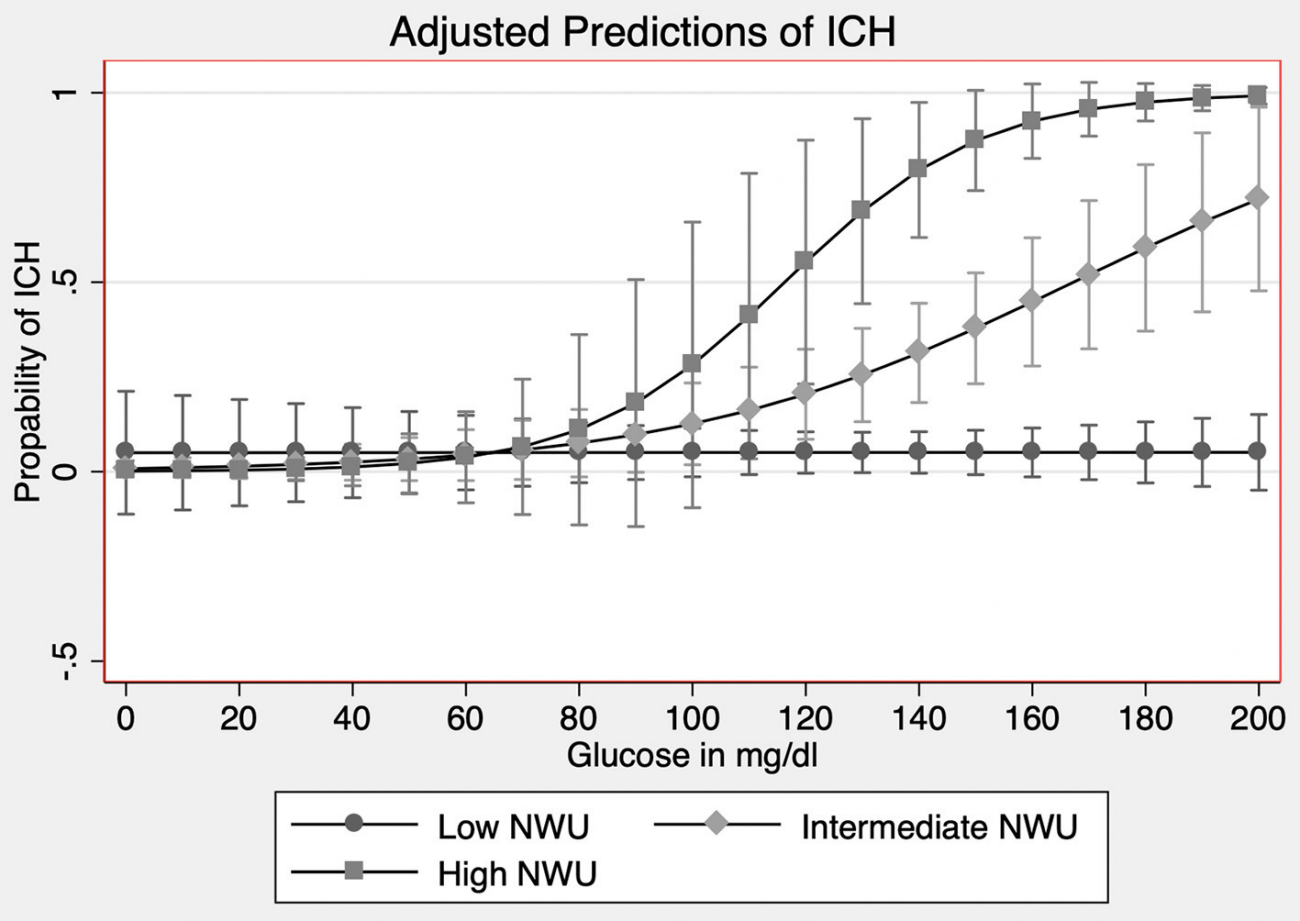
Interaction analysis of BGL and NWU on the occurrence of ICH. Occurrence of secondary intracerebral hemorrhage (ICH, *y*-axis) according to trichotomized NWU (net water uptake; low NWU: <7%; intermediate NWU: 7–12%; high NWU: >12%) and BGL (baseline glucose level; glucose in mg/dl, *x*-axis). The interaction term between NWU and BGL was significant.

Data were tested for normality and homogeneity of variance using histogram plots and Kolmogorov–Smirnov-tests. Absolute and relative frequencies are given for categorical data. Median and interquartile range (IQR) are given for univariable distribution of metric variables. Patients with ICH vs. without ICH were compared by Mann–Whitney *U*-test for metric outcome variables and by chi-square test for categorical outcome variables (Table 1). Kappa statistic and calculated Cohen's *k* were used for interrater reliability measurement. ROC analysis was assessed to analyze the diagnostic performance for prediction of ICH after successful MT using increasing discrimination thresholds of independent predictors and cutoffs determined according to the Youden index (24, 25). The association between clinical and radiological parameters and ICH in patients after successful MT was assessed by univariate logistic regression analysis. For multivariate logistic regression analysis, a model with forward selection was used to identify significant variables for developing ICH (inclusion criterion: *P*-value of the score test ≤ 0.05, exclusion criterion: *P*-value of the likelihood ratio test >0.1) (Table 2). Given for selected variables are odds ratio (OR) with 95% CI and *P*-value of likelihood ratio test. For non-selected variables, *P*-value of score test is displayed. The impact of BGL on the association of NWU and occcurence of ICH was tested using logistic regression analysis. The OR for NWU increase was assessed for patients with five different levels of blood glucose based on the relative distribution of BGL (Table 3). Finally, to further analyze the relationship of early edema formation and BGL, we trichotomized patients into three groups based on the distribution of NWU within the patient cohort. The interaction term for trichotomized NWU and BGL was calculated and plotted (Figure 2). A statistically significant difference was accepted at a *P*-value of <0.05. No adjustment for mutlitple testing was performed as the analyses being explorative in nature. Analyses were performed using MedCalc (version 11.5.1.0; Mariakerke, Belgium) and Stata/SE 13.0 (StataCorp, College Station, TX, USA).

**Table 1:** Comparison of demographic, clinical, and radiological characteristics between patients with intracerebral hemorrhage and those with no intracerebral hemorrhage after successful mechanical recanalization.

**Baseline characteristics**	**All** ** (*n* = 107)**	**Without ICH** ** (*n* = 70)**	**With ICH** ** (*n* = 37)**	***P*-value**
Age (years), median (IQR)	76 (65.0; 81.0)	75 (64.8; 80.0)	79 (69; 84)	0.074
Female, *n* (%)	55 (51.4)	34 (48.6)	21 (56.8)	0.420
Hypertension, *n* (%)	60 (56.1)	36 (51.4)	24 (64.9)	0.183
Diabetes mellitus, *n* (%)	20 (18.7)	14 (20.0)	6 (16.2)	0.633
Atrial fibrillation, *n* (%)	39 (36.4)	19 (27.1)	20 (54.1)	0.006
Smoking, *n* (%)	15 (16.9)	10 (18.2)	5 (14.7)	0.670
Dyslipidemia, *n* (%)	23 (21.5)	16 (22.9)	7 (18.9)	0.637
Blood glucose (mg/dl), median (IQR)	135 (110.25; 178.5)	126 (106.5; 147)	177 (127; 221.75)	<0.001
**CT parameters, median (IQR)**				
• ASPECTS	8 (6; 9)	8 (6.0; 9.0)	7 (5.5; 8.0)	0.02
• Collateral score	2 (1.0; 3.0)	2 (2.0; 3.0)	1 (1.0;2.0)	<0.001
• Net water uptake (NWU)	0.04 (0.00; 0.1)	0.05 (0.03; 0.09)	0.13 (0.1; 0.16)	<0.001
**Stroke cause**, ***n*** **(%)**				0.054
• Cardioembolic	56 (52.3)	33 (47.1)	23 (62.2)	
• Large-artery atherosclerosis	42 (39.3)	28 (40.0)	14 (37.8)	
• Others	9 (8.4)	9 (12.9)	0 (0)	
**Procedure process and results**				
• General anesthesia, *n* (%)	75.0 (70.1)	52 (74.3)	14 (37.8)	0.193
• Intravenous thrombolysis, *n* (%)	63 (58.9)	26 (37.1)	19 (51.4)	0.250
• OTI (h), median (IQR)	2:35 (1:06; 3:44)	2:24 (0:59; 3:42)	2:40 (2:07; 3:51)	0.438
• ITR (h), median (IQR)	1:47 (1:23; 2:03)	1:40 (1:19; 1:58)	1:53 (1:33; 2:10)	0.243
• Passes of retriever	2 (1; 2)	2 (1; 2)	2 (1; 3)	0.071
• mTICI (2b, 3 grouped), *n* (%)	107 (100)	70 (100)	37 (100)	0.486
**Clinical parameters**				
• NIHSS on admission	13 (12; 19)	15 (12.0; 18.0)	19 (16.0; 20.0)	0.005
• NIHSS after 24 h	3 (5; 20)	10 (4.0; 16.0)	19 (13.0; 34.5)	<0.001
• NIHSS at discharge	1.5 (3; 14)	5 (1.0; 9.8)	14 (7.5; 18.5)	<0.001
**90-day mRS, median (IQR)**	4 (3.2; 4.0)	3 (2.5; 3.6)	5 (4.3; 5.3)	<0.001
• 0–1, *n* (%)	28 (28.0)	25 (37.3)	3 (9.1)	0.003
• 2–3, *n* (%)	15 (15)	11 (16.4)	4 (12.1)	0.572
• 4–6, *n* (%)	55 (51.4)	29 (44.6)	26 (78.8)	0.001

*ICH, intracerebral hemorrhage; IQR, interquartile range; TICI, thrombolysis in cerebral infarction; NIHSS, National Institutes of Health Stroke Scale; TICI, thrombolysis in cerebral infarction; INR, international normalized ratio*.

**Table 2:** Multivariable analysis of predictors of secondary hemorrhage after successful mechanical recanalization.

**Serum glucose (mg/dl)**	**OR for ICH**	**95% CI**	***P*-value**
Net water uptake (NWU; per %)	2.31	1.33–3.73	<0.001
Collateral score (ref: poor)	0.05	0.008–0.27	0.0007
Blood glucose (mg/dl)	1.02	1.00–1.04	0.01

*Non-significant independent variables are not displayed. Given for selected variables are odds ratios (OR) with 95% confidence interval (CI) and P-value of likelihood ratio test*.

*ICH, intracerebral hemorrhage*.

**Table 3:** The association of NWU and occurrence of secondary hemorrhage after successful mechanical recanalization, mediated by different baseline serum glucose levels.

**Serum glucose (mg/dl)**	**OR for NWU (per %)**	**95% CI**	***P*-value**
<120	1.34	1.00–1.79	0.05
<134 (median)	1.39	1.09–1.78	0.008
>134	1.63	1.24–2.13	0.0005
>150	1.87	1.22–2.87	0.004
>170	2.08	1.10–3.92	0.02

*Odds ratios (OR) for net water uptake (NWU) given for selected levels of serum blood glucose with 95% confidence interval (CI) and P-value of likelihood ratio test*.

## Results

### Patients

**Figure 3:**
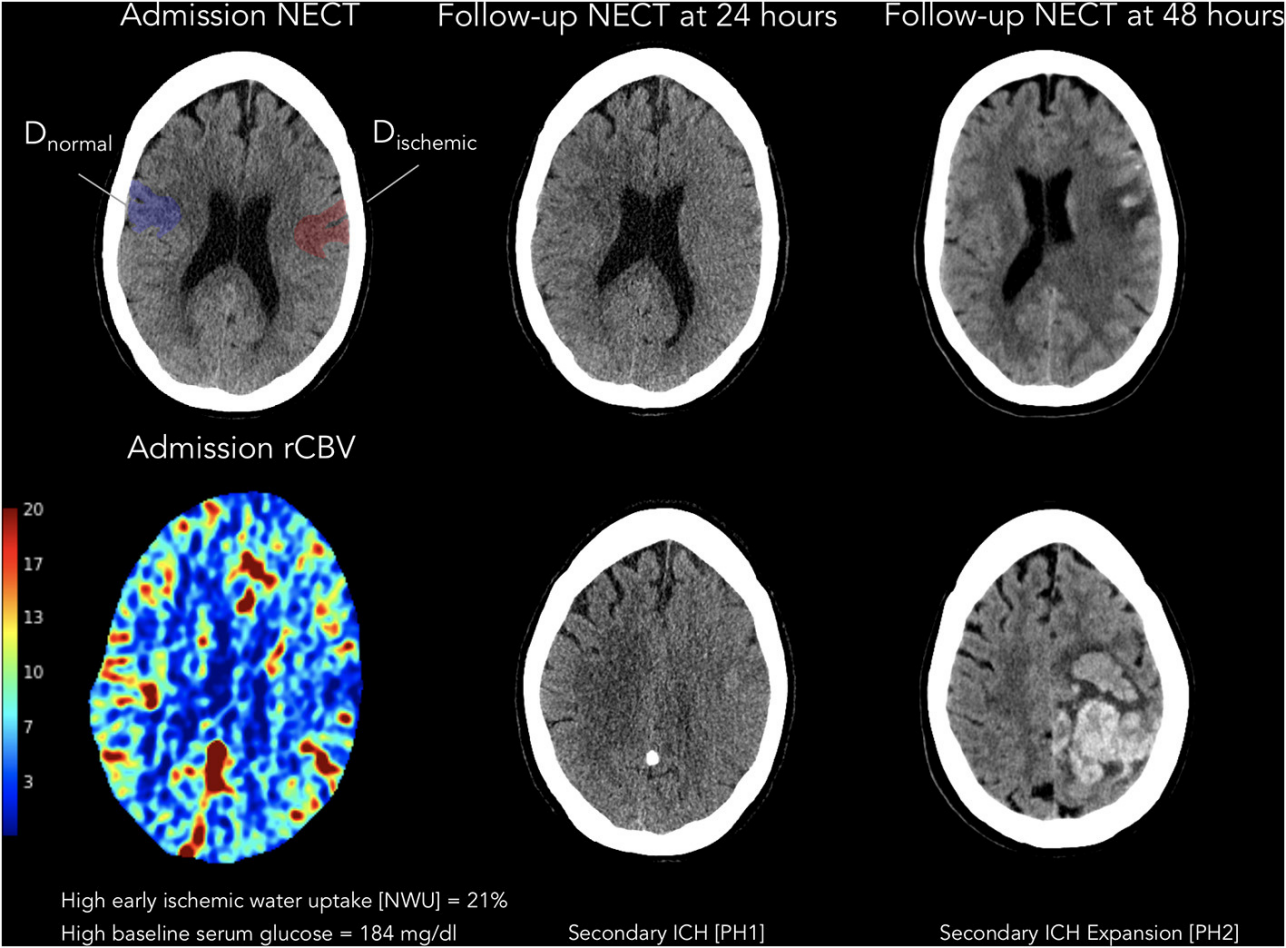
Illustrative example of a patient with baseline glucose and water uptake and secondary ICH after successful thrombectomy. Illustration of a patient with high early baseline blood glucose and high ischemic water uptake, with subsequent secondary intracerebral hemorrhage after thrombectomy. On the left, patient admission images are displayed with admission non-enhanced CT and ROIs for ischemic hypoattenuation (*D*_ischemic_) and on the contralateral side (*D*_normal_) as well as relative CBV (rCBV). The rCBV map (ml per 100 ml) is inferred from a quantitative assessment of the partial volume averaging in each pixel. In the middle, follow-up non-enhanced CT images at 24 h are displayed with secondary intracerebral hemorrhage (parenchymal hematoma; grade 1). On the right, follow-up non-enhanced CT images at 48 h are displayed with secondary intracerebral hemorrhage (parenchymal hematoma; grade 2). rCBV, relative cerebral blood volume; NECT, non-enhanced CT; PH1, parenchymal hematoma grade 1; PH2, parenchymal hematoma grade 2.

**Figure 4:**
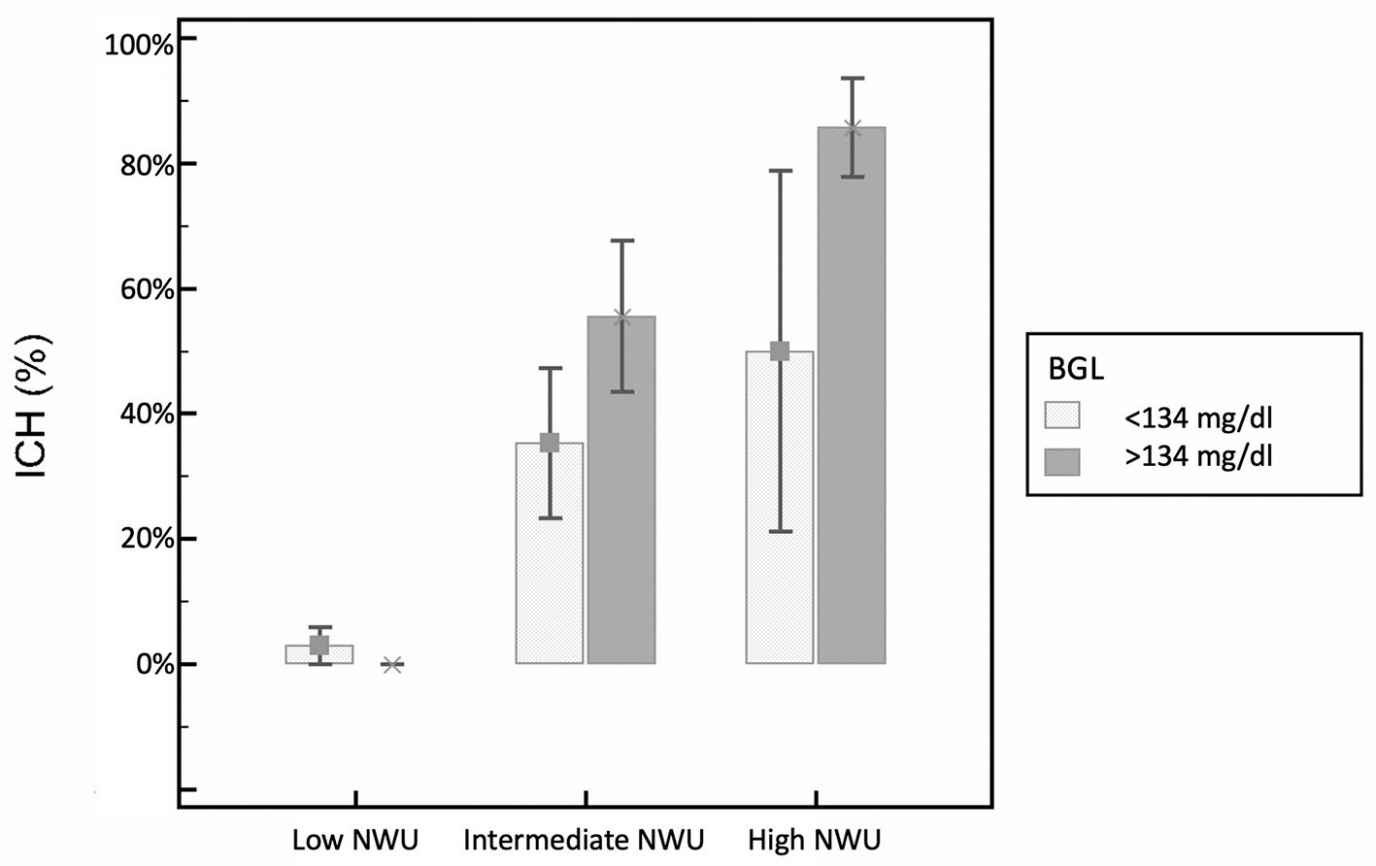
Relationship of BGL and NWU on the occurrence of ICH. Occurrence of secondary intracerebral hemorrhage (ICH) according to baseline NWU (net water uptake) and BGL (baseline glucose level), separately for patients with low and high BGL (based on the median BGL) and trichotomized NWU based on relative distribution of NWU within the patient cohort (low NWU: <7%; intermediate NWU: 7–12%; high NWU: >12%).

Out of 336 patients, 107 consecutive patients were included according to the inclusion criteria [median age 76 years (IQR: 65.0–81.0) and 51.4% females] with 63 patients (58.9%) having received intravenous thrombolysis before MT. An example is illustrated in Figure 3. Thirty-seven patients (34.6%) had an ICH within 24 h after MT according to the Heidelberg Bleeding Classification with sICH identified in 19 patients (17.8%). Admission NIHSS was significantly higher in patients with ICH (*P* = 0.005). Also, both significantly higher NIHSS after 24 h and at discharge were observed in patients with ICH (*P* < 0.001). Further patient characteristics are summarized in Table 1. A history of diabetes was present in 20 (18.7%) patients with no statistical differences between patients with and without ICH (*P* = 0.63). The median blood glucose level at admission was 134 mg/dl (IQR: 110.0–178.5) with significant higher levels in patients with ICH 177 mg/dl (IQR: 127–221.75) vs. 126 mg/dl (IQR: 106–147) in patients without ICH (*P* < 0.001). CT-based derived parameters of quantitative NWU and collateral score were significantly different for both groups with a higher NWU in patients with ICH (13%; IQR: 11–16%) and lower collateral score (1; IQR: 1–2) vs. lower NWU (5%; IQR: 3–9%) and higher collateral score (2; IQR: 2–3) in patients without ICH. Baseline Alberta Stroke Program Early CT (ASPECTS) was statistically different between both groups, with ASPECTS 7 in patients with ICH (IQR: 5.5–8) and ASPECTS 8 in patients without ICH (IQR: 6–9, *P* = 0.02). Unfavorable clinical outcome at 90 days (mRS score 4–6) was higher in patients with ICH (78.8 vs. 44.6%, *P* = 0.001). Excellent clinical outcome (mRS score 0–1) at 90 days was higher in patients without ICH (37.3 vs. 9.1% with ICH, *P* = 0.003). Figure 4 illustrates the relationship of BGL and NWU on the occurrence of ICH.

### Prediction of ICH

**Figure 5:**
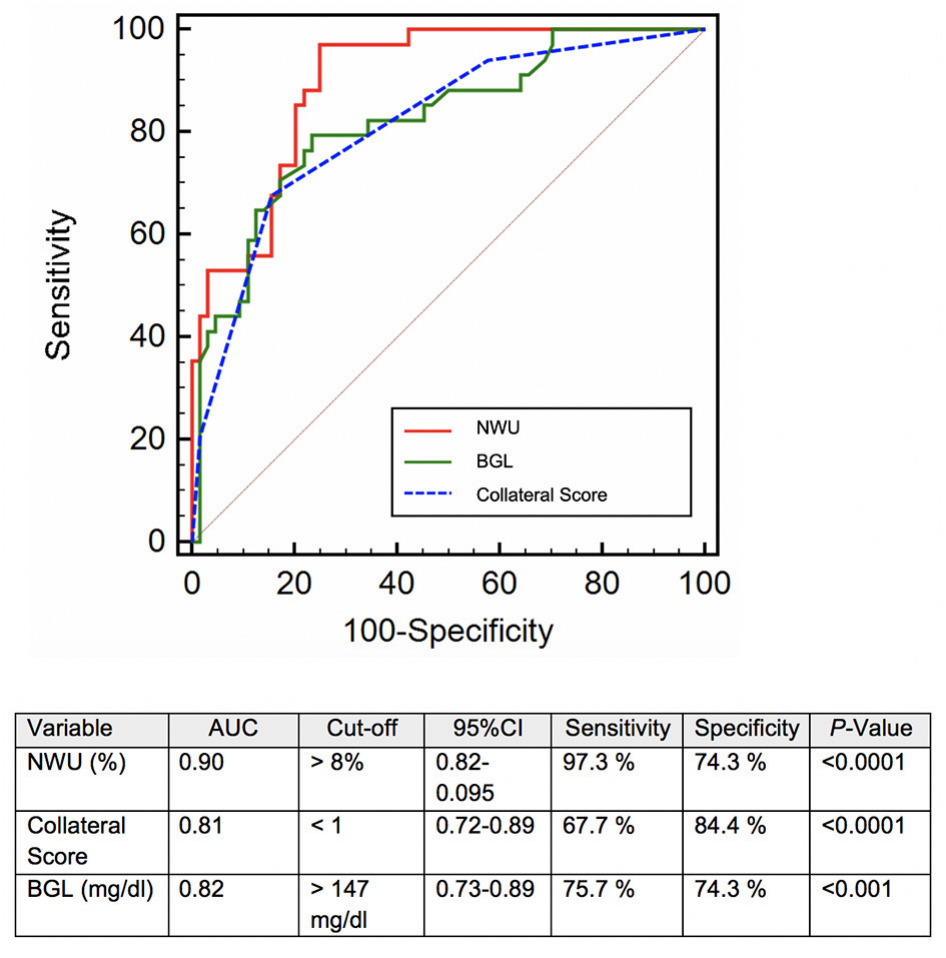
Receiver operating curve (ROC) analysis for the prediction of secondary hemorrhage after successful mechanical recanalization. ASPECTS, Alberta Stroke Program Early CT Score; AUC, area under the curve; 95% CI, 95% confidence interval; NWU, net water uptake; BGL, baseline glucose level.

Univariate ROC analysis was performed to identify the diagnostic accuracy of independent variables of univariate logistic regression. NWU with an optimal cutoff above 8% predicted ICH with the highest discriminative power [area under the curve (AUC): 0.90, 95% CI: 0.82–0.95; specificity 74.3%, sensitivity 97.3%, *P* < 0.0001], followed by collateral score with an optimal cutoff below 1 (AUC: 0.81, 95% CI: 0.72–0.89; specificity 84.4%, sensitivity 67.7%, *P* < 0.0001) and admission serum glucose with an optimal cutoff above 147 mmol/L (AUC: 0.82, 95% CI: 0.73–0.89; specificity 74.3%, sensitivity 75.7%, *P* < 0.011; Figure 5).

Logistic regression analysis was performed to assess the association between various clinical and radiological parameters and the incidence of ICH after successful MT. At univariate logistic regression analysis, ICH was predicted by higher admission serum glucose (OR 1.01 per median, 95% CI: 1.00–1.02, *P* = 0.048), lower collateral score (OR 0.37 per median, 95% CI: 0.20–0.67, *P* < 0.001), high admission NIHSS (OR: 1.08, 95% CI: 1.01–1.15, *P* = 0.027), low ASPECTS (OR: 0.78, 95% CI: 0.61–0.99, *P* = 0.041), and higher early NWU (OR: 1.56, 95% CI: 1.31–1.86, *P* < 0.0001). In multivariable logistic regression analysis, a higher degree of NWU (adjusted OR 2.31 per %, 95% CI: 1.33–3.73, *P* = 0.0006), a lower collateral score (adjusted OR 0.05 per median, 95% CI: 0.008–0.27, *P* = 0.0007), a lower ASPECTS (OR: 2.71, 95% CI: 1.31–5.61, *P* = 0.007), and a higher BGL (adjusted OR 1.02 per median, 95% CI: 1.00–1.04, *P* = 0.01) were identified as independent predictors of ICH after successful MT (Table 2). Higher admission NIHSS was not significantly associated with ICH (OR: 1.06, 95% CI: 0.94–1.2, *P* = 0.35).

### Interaction of NWU and BGL

We tested how the likelihood for ICH by increasing NWU is associated with concordant increasing levels of BGL. For patients with lower BGL (<134 mg/dl, median), higher NWU was associated with a 1.39-fold likelihood for ICH, while for patients with higher BGL (>134 mg/dl), NWU increase was associated with 1.63-fold likelihood for ICH. For patients with very high BGL (>170 mg/dl), a NWU increase was associated with a 2.08-fold likelihood for ICH (Table 3). Finally, NWU was trichotomized into low (<7%), intermediate (7–12%), and high NWU (>12%). A higher trichotomized NWU was significantly associated with increased likelihood of ICH (OR: 9.41, 95% CI: 3.81–23.27, *P* < 0.001). The interaction term between NWU and BGL was significant (OR: 1.03, 95% CI: 1.01–1.06, *P* = 0.04; Figure 2).

### Subanalysis for Patients With Intermediate and High NWU but Low BGL

Finally, patients with intermediate and high NWU (>7%) but low BGL (<134 mg/dl, median) were investigated. Comparing these patients to patients with higher BGL (>134 mg/dl), there were no significant differences in age, ASPECTS, NIHSS, or time from onset to imaging. However, patients with intermediate and high NWU but low BGL showed a significantly lower collateral score (1.4 vs. 2.2, *P* = 0.02).

## Discussion

Higher admission BGL as an independent predictor for developing an ICH after successful ET in AIS paired with the association between ischemic edema is the main finding of our study. Furthermore, the effect of NWU on the occurrence of ICH was increased in patients with higher BGL suggesting a specific interrelation between early edema formation, as a sign of blood–brain barrier injury, and BGL as a potential “accelerator” of blood–brain barrier injury. This finding was accompanied by elevated early ischemic edema and poor collateral score as a second independent predictor for ICH. BGL and NWU showed a significant interaction indicating that the slopes for likelihood of ICH with increasing BGL differ significantly according to the degree of NWU, as illustrated in Figure 2. In patients with very low early NWU, BGL increase did not alter the risk of ICH, while a BGL increase in patients with higher NWU resulted in a significant increase in likelihood for ICH. The specific interaction between early edema formation and serum glucose levels and its impact on the occurrence of ICH has not yet been described and might be a hint of a specific pathophysiological association. It has been observed that elevated levels of BGL are associated with aggravated edema formation (13), as a sign of blood–barrier injury, and that elevated levels of early edema formation increased the risk of secondary hemorrhage (12). Hence, the coexistence of high BGL and high NWU might be a constellation of very high risk for ICH and should be a hint for clinicians to indicate stricter monitoring and consider adjustment of glucose levels. Moreover, the findings could enrich for patients to study experimental treatments with antiedematous drugs, such as glyburide. Glyburide, an antidiabetic drug, is an inhibitor of the sulfonylurea receptor 1 and transient receptor potential melastatin 4 (SUR1-TRPM4) (26). The application of glyburide is safe and feasible and has been described for preventing edematous brain edema (27). In previous pre-clinical studies using rodent models with malignant edema, inhibition of SUR1 resulted in lower ischemic lesion volume, reduced mortality, and better functional outcome (28). Supporting these pre-clinical data, retrospective analyses on patients with diabetes and AIS observed that patients with medication of sulfonylurea drugs had an improved clinical outcome and lower rates of hemorrhagic transformation (HT) (29).

The nature of the association between acute elevated levels of admission glucose and increased risk of ICH has already been investigated in animal models of AIS as well as in both retrospective and prospective clinical imaging studies (7, 30–33). Previous studies evaluating the risk and rates of ICH have been hampered by the lack of consensus definitions for bleeding events (31). The recently introduced Heidelberg Bleeding Classification provides a standardized and reproducible tool and basis for evaluating further treatment strategies (34). Nevertheless, our study is methodically limited to the important fact that a systematic approach in classifying symptomatic ICH is missing which should be addressed in future clinical studies with larger patient cohorts. The quantitative stratification of ischemic brain edema *via* NWU demonstrates the direct relation of hypoattenuation in CT to the percentage of volume of water uptake and has been validated with excellent sensitivity and reproducibility since (19, 20, 35). Recent findings of our study group give two major explanatory reasons for poor clinical outcome after MT and the risk of an ICH. Firstly, poor clinical outcome has been associated with increased levels of ischemic edema and further associated with poor collateral score and elevated BGL (12, 13, 36). Secondly, collaterals and the degree of NWU both mediate tissue vulnerability and the risk for an ICH (12). In line with this, the recently published study of Hao et al. also reported poor collateral circulation as an independent predictor for ICH (37). Yet, the relationship of NWU and hyperglycemia with respect to the occurrence of ICH after successful MT remains unknown. The pathophysiology adds support to our hypothesis as both elevated BGL and ischemic edema share a common pathway of impaired blood–brain barrier (BBB) with the risk of increased tissue vulnerability: ICH after MT occurs due to a reperfusion syndrome from rupture of necrotic vessel walls and increased BBB permeability due to prolonged ischemia (38, 39). By mediating both oxidative stress and inflammation response in vessel walls, hyperglycemia is also associated with increased reperfusion injury (40–42). The aggravated breakdown of the BBB results in further edema formation and increased infarct volume (8, 43). Thorén et al. have investigated the impact of hyperglycemia on admission as an independent risk factor for cerebral edema in patients with AIS treated with IVT and obtained the extent of ischemic edema with a visual rating system (11). This study is in concordance with our previous findings that proved that the degree of early edema formation using a quantitative imaging biomarker is a risk factor for secondary ICH, possibly potentiated by increased levels of BGL (12, 38, 39). Taken all together, these independent variables may provide a closed loop that provides a target for therapeutic intervention to disrupt the cycle. Further clinical studies analyzing the mediated effect of NWU on the risk of ICH by increased levels of BGL are therefore needed. Also, potential beneficial treatment effects from the control of hyperglycemia in patients with AIS have been described numerously (44), yet established treatment methods are still under investigation. Although results from experimental studies support a causal relationship between hyperglycemia and poor functional outcome after stroke, multicenter trial data presented in the SHINE, GIST-UK, or THIS trial do not yet support intervention with insulin (45–47). In the future, clinical trials might consider combining reperfusion with further adjuvant treatment in patients with AIS and elevated levels of admission glucose. Yet, standardized methods to monitor possible effects on ischemic tissue edema are still missing. In this respect, quantitative NWU might provide a feasible imaging biomarker to monitor the effects of these drugs in prospective clinical trials, subject to the condition of a confirmed mediated effect of NWU by BGL (26, 27, 48).

Several limitations of our study deserve attention. First, for the assessment of NWU measurements, ROIs were drawn by a quantitative edge detection tool and boundaries adjusted manually if necessary, for example, in cases of anatomical asymmetry which may hamper NWU quantification. However, semiautomatic methods of Hounsfield unit value thresholding have been used in our previous studies with excellent reproducibly and partially mitigate a method bias (17, 21). Interrater reliability may further help to augment the accuracy of NWU measurements in the future. Additionally, the lack of information on the longitudinal course of glucose levels upon FU-NECT and the undocumented use of blood glucose-lowering drugs limit the generalizability of our results. Furthermore, the purpose of this study was to predict secondary ICH without further differentiating into symptomatic or asymptomatic ICH. As it has been observed in both retrospective and prospective studies that asymptomatic ICH is also associated with worse functional outcome, we nevertheless consider our observation to be of importance (4–6). In light of these findings, it is relevant to investigate the risk factors for any ICH, to finally improve the prevention of ICH in patients undergoing MT. Finally, according to the retrospective nature of the study, no causality assumptions can be inferred from the obtained data.

## Conclusions

Our study confirmed that higher BGL increased the likelihood for ICH, but depends on the degree of early ischemic edema. Although a causal relationship between NWU and higher BGL and ICH remains speculative and more data are needed, specific interactions between BGL and NWU may be tested as a further therapeutic target.

## Data Availability Statement

The data analyzed in this study is subject to the following licenses/restrictions: According to the institution's strict data security regulations the entire datasets are not publicly available. Requests to access these datasets should be directed to jawed.nawabi@charite.de.

## Ethics Statement

The studies involving human participants were reviewed and approved by Ärztekammer Hamburg WF-035/18. Written informed consent for participation was not required for this study in accordance with the national legislation and the institutional requirements.

## Author Contributions

All authors listed have made substantial, direct and intellectual contribution to the work and approved it for publication.

## Conflict of Interest

The authors declare that the research was conducted in the absence of any commercial or financial relationships that could be construed as a potential conflict of interest.
